# Unravelling the Potential Regulatory Role of Blood lncRNA on Swine Immunity

**DOI:** 10.3390/cells15181681

**Published:** 2026-09-17

**Authors:** Teodor Jové-Juncà, Carles Hernández-Banqué, Daniel Crespo-Piazuelo, Olga González-Rodríguez, Raquel Quintanilla, Maria Ballester

**Affiliations:** Animal Breeding and Genetics Program, Institute of Agrifood Research and Technology (IRTA), Torre Marimon, 08140 Caldes de Montbui, Barcelona, Spaincarles.hernandez@irta.cat (C.H.-B.); olga.gonzalez@irta.cat (O.G.-R.)

**Keywords:** transcriptomics, pig, coexpression, non-coding RNA, RNA-RNA interactions, immune system

## Abstract

**Highlights:**

**What are the main findings?**

- A major lncRNA-associated network comprising 18 lncRNAs and 206 genes was identified in the porcine blood transcriptome.
- Several putative RNA-RNA interactions were identified between lncRNAs and immune-related candidate genes.

**What are the implications of the main findings?**

- Similar network architectures potentially linked to immunity in Duroc blood and Meishan spleen suggest that lncRNAs may contribute to shared immunoregulatory processes across tissues.
- Our findings highlight candidate lncRNA-mediated regulatory mechanisms underlying porcine immunity and potential RNA-RNA interactions for future validation.

**Abstract:**

Long non-coding RNAs (lncRNAs) are regulatory RNA molecules involved in numerous biological processes, including immune response. Although their functions remain difficult to characterize, lncRNAs have been associated with both productive and immune traits in pigs. Because immune parameters have been proposed as indicators of immunocompetence, studying lncRNA regulation in immune-related tissues may contribute to improving pig robustness and disease resistance. In this study, whole-blood RNA-seq data from 255 commercial 60-day-old Duroc pigs were used to identify lncRNA-associated immune mechanisms. Co-expression networks were constructed from highly correlated lncRNAs and other genes, followed by functional enrichment analyses. Associations between network genes and 15 immune-related phenotypes were evaluated, and putative RNA–RNA interactions were characterized. The largest network identified was enriched for immune pathways, particularly antiviral response, and included key transcription factors such as *IRF7*, *STAT1*, *STAT2*, and *ZNFX1*. Four genes within this network (*ASAP3*, ENSSSCG00000036342, *RNF24*, and *P2RY13*) were in silico predicted to interact with the same domain of lncRNA ENSSSCG00000055468. Additional networks were associated with V(D)J recombination, cytokine-mediated signalling, and other immune functions. A second putative RNA–RNA interaction was identified between lncRNA ENSSSCG00000058144 and *RHEX*. These findings reveal potential lncRNA-mediated regulatory mechanisms underlying porcine immunity and provide candidate RNA-RNA interactions that require future validation.

## 1. Introduction

The advent of cheaper sequencing technology has allowed the study of transcriptomics to a depth and scale unseen before. It is well known that, although a great portion of the mammalian genome is transcribed, a significant portion of transcripts do not encode for any protein [1]. While small non-coding RNA molecules have generally well-defined functions and are classified accordingly, the functions of long non-coding transcripts are less well characterized. Non-coding transcripts longer than 200 bp are classified as long non-coding RNAs (lncRNAs) [2]. These lncRNAs are not as abundant as coding transcripts [3] and usually have expression patterns that are highly dependent on tissue, external conditions and developmental stage [4]. Furthermore, lncRNA sequences are generally not conserved between species [2]. The best characterized properties of lncRNAs relate to their mechanisms of action, which are predominantly regulatory. They function as miRNA sponges, are post-processed into miRNAs [5], and modulate chromatin architecture and enhancer activity through interactions with transcription factors, gene-activating complexes, and biomolecular condensates [2]. At the biological level, lncRNAs have been implicated in a variety of processes, such as V(D)J recombination [6], regulation of cytokine expression [7], inflammatory pain responses [8], growth hormone and prolactin production [9] and glucose metabolism [10,11], among others in humans and model species [2]. Despite all the mechanisms described above, the specific functions of many lncRNAs remain unknown, and their characterization continues to be an active area of research.

Several studies have identified lncRNAs associated with economically important traits in livestock species (reviewed in Lagarrigue et al. [12]). In pigs, Esteve-Codina et al. [13] reported lncRNAs associated with pathways related to gamete production and lipid metabolism in gonads. More recently, Zhao et al. [14] described lncRNAs that were differentially expressed in the liver and small intestine of pigs with divergent feed efficiency.

The role of porcine lncRNAs in immunity has also been characterized. This is particularly relevant because immune parameters have been proposed as candidate selection criteria to improve disease resistance and animal robustness [15]. Wu et al. identified the lncRNA FUT3-AS1 as a host-regulator associated with *Escherichia coli F18*-induced diarrhea [16]. In addition, Shi et al. [17] investigated the relationship between immune-related genes and lncRNAs in the spleen of Meishan pigs. Furthermore, Zhang et al. profiled the first transcriptomic landscape of lncRNAs and mRNAs in LPS-induced porcine PBMCs [18]. More recently, Li et al. studied the transcriptional differences (mRNAs and lncRNAs) between pre- and post-swine fever vaccination Min and Large White pigs, describing immune-related pathways that could contribute to the observed differences in disease resistance between both swine breeds [19].

Identifying biomarkers associated with pig immunity could enhance genetic gain by providing measurable molecular indicators that are directly linked to the underlying genetic architecture of immune traits. In this context, the expression of lncRNAs and their underlying genetic regulation represent strong biomarker candidates due to their regulatory role in multiple immune processes. The characterization of gene regulatory and coexpression networks has been reported to aid in biomarker identification [20,21]. Therefore, the characterization of gene networks regulated by lncRNAs could provide effective biomarkers for improving the genetic selection of immune traits.

Overall, these studies not only explored the functional relevance of lncRNAs and their associations with economically important and immune-related traits, but have also contributed to the identification and annotation of novel porcine lncRNAs [22]. However, to date, immune-related lncRNAs associated with global immunocompetence have not been investigated in peripheral blood from healthy pigs. This is particularly relevant, as the transcriptomic profile of peripheral blood has been shown to correlate with immunocompetence in pigs [23,24].

In the present work, we analyzed the whole-blood transcriptome of 255 commercial Duroc pigs to identify lncRNAs associated with immune capacity, using a network-based approach to investigate pathways related to immunity, as well as to explore potential RNA-RNA interactions.

## 2. Materials and Methods

### 2.1. Animal Material

The present study was conducted using a commercial Duroc population comprising 255 animals (129 females and 126 males). Animals were raised in 6 consecutive batches (between 37 and 46 individuals per batch) on the same farm and fed an ad libitum cereal-based diet. Blood samples were collected at 60 ± 8 days of age. Blood samples were extracted from the external jugular vein into Vacutainer tubes with or without anticoagulants (Sangüesa S.A., Cornellà del Llobregat, Spain) for immunophenotyping, and into Tempus^TM^ Blood RNA tubes (Thermo Fisher Scientific, Alcobendas, Spain) for RNA extraction. Samples were transported to the laboratory on ice blocks and stored at −20 °C for immunophenotyping and −80 °C for RNA extraction.

In situ physical assessment indicated that pigs were physically healthy, which was subsequently confirmed by blood parameter analysis. The population showed no signs of immunosuppression, subclinical infection, acute stress, or acute/chronic inflammatory responses, as their immunocompetence parameters fell within the expected 95% confidence interval [25].

### 2.2. Immunophenotyping

A total of 15 immune traits, selected from previous studies carried out in the same population [26,27], were considered for the analysis. Parameters representative of both innate and adaptive immunity were selected, including immunoglobulin and acute-phase protein concentrations, leukocyte and peripheral mononuclear blood cell (PBMC) subpopulations, phagocytic measures, and hematological parameters.

Total concentration of immunoglobulin G (IgG) and M (IgM) and C-reactive protein (CRP) were measured by ELISA (Bethyl Laboratories Inc., Bionova, Madrid, Spain and Abcam plc, Cambridge, United Kingdom, respectively) from plasma and serum samples, respectively. Haptoglobin concentration was measured in serum samples by colorimetric assay (Tridelta Development Limited, Maynooth, Ireland). Plates were read using an ELISA plate reader (Bio-Rad, Hercules, CA, USA) and analyzed using the Microplate Manager 5.2.1 software (Bio-Rad).

The percentage of phagocytic cells was measured by incubating blood samples with fluorescein-labelled opsonized *E. coli* (BD Pharmigen, San Agustín del Guadalix, Spain) and then read with a MACSQuant Analyzer 10 Flow cytometer (Miltenyi Biotec GmbH, Bergisch Gladbach, Germany) using the MACSQuantify software v2.6 (Miltenyi Biotec GmbH).

Lymphocyte and neutrophil counts, as well as mean corpuscular hemoglobin, were determined by hemogram analysis using a CELL-DYN^®^ 3700 analyzer (Abbott, Madrid, Spain) at Laboratory Echevarne (Barcelona, Spain).

Finally, several cellular subpopulations of PBMC were measured by flow cytometry using the MACSQuant Analyzer 10 Flow cytometer (Miltenyi Biotec GmbH) and the Flowlogic™ software v7.3 (Inivai Technologies, Melbourne, Australia). In the present study, the following cell subsets were quantified: cytotoxic (CTL) T cells (CD3^+^ CD4^−^ CD8^+^), memory T-helper cells (CD3^+^ CD4^+^ CD8^+^) and naïve T cells (CD3^+^ CD4^−^ CD8^−^), as well as total T cells (CD3^+^), B cells (CD21^+^) and natural killer cells (CD3^−^ CD21^−^ CD8^+^). The antibodies used were FITC Mouse Anti-Pig CD8α (76-2-11 clone), Alexa Fluor^®^ 647 Mouse Anti-Pig CD4α (74-12-4 clone), PE-Cy™7 Mouse Anti-Pig CD3ε (BB23-8E6-8c8 clone) (BD Biosciences, San Agustín del Guadalix, Spain) and PE Mouse Anti-Pig CD21 (BB6-11C9.6 clone) (Southern Biotech, BioNova). γδ T cells were also measured with the specific antibody APC Rat Anti-Pig γδ T Lymphocytes (MAC320 clone) (BD Biosciences).

All phenotypes were tested for possible cofactors: sex, animal batch and date of measurement. Corrections were performed with the lm function of base R [28]. A table containing descriptive statistics of the considered traits as well as normalization method and relevant cofactors can be found in Supplementary Table S1.

### 2.3. RNA Sequencing and lncRNA Identification

As described in Jové-Juncà et al. [24], whole-blood RNA was extracted using Tempus^TM^ Spin RNA Isolation Reagent Kit (Thermo Fisher Scientific). Total RNA concentration and quality were assessed using a NanoDrop ND-1000 spectrophotometer and a Fragment Analyzer (Agilent Technologies Inc., Santa Clara, CA, USA).

Libraries were prepared using the Stranded Total RNA with Ribo-Zero Plus rRNA depletion (Illumina, San Diego, CA, USA) and were sequenced using an Illumina NovaSeq6000 platform at Centro Nacional de Análisis Genómico (CNAG-CRG, Barcelona, Spain) with a depth of >55 M paired-end (2 × 150 bp) reads. Quality was assessed with FastQC software v0.12.1 [29]. Mapping was performed against the reference genome (Sscrofa11.1 assembly) using STAR v2.75.3a [30] and then quantified using RSEM v1.3.0 [31]. On average, a total of 161.5 million reads per individual were obtained, of which 44.06% mapped to exonic regions, 47.09% to intronic regions, and 8.4% to intergenic regions.

Counts were then normalized using the trimmed mean of M-values methodology using the EdgeR 3.23 package [32], extracting the log counts per million. Only genes with cpm above 10/minimum library size in millions (i.e., cpm > 0.69) in more than 5% of the samples were retained. Expression levels were then corrected by sex and animal batch per gene using the lm function of base R [28]. LncRNAs were identified using the Ensembl Biomart tool [33]. To further annotate potential interactions between lncRNA and nearby genes, the FEELnc classifier module from the FEELnc v0.2.1 software [34] was used, with the Sscrofa11.1 genome assembly and annotation as reference. This tool provided a list of possible lncRNA–gene interactions and classified them according to strand, sense, and relative position (intronic, exonic, or intergenic).

### 2.4. Coexpression and Interaction Analyses

To investigate relationships among gene expression levels, the Partial Correlation with Information Theory (PCIT) algorithm was used in R 4.5.3 using the PCIT 1.5-3 package [35]. The full table of expressed genes and immune phenotypes was used as input. To study potential gene networks including lncRNA and immune traits, the following correlation cases were selected from the list of direct correlations: (1) interactions containing at least one lncRNA with a correlation weight greater than 0.9; (2) interactions containing a gene already involved in one of the previous interactions, also with a correlation above 0.9; and (3) interactions including one of the genes from the previous two groups and an immune trait, with a correlation weight greater than 0.3. A lower correlation threshold was selected for interactions including traits, as the relationship between gene expression and complex traits is expected to be less close than between two potentially interacting genes.

The selected interactions between two genes were classified as *cis* and *trans* based on their relative genomic positions. Genes located less than 1 Mb apart were considered to potentially exert a local effect on each other’s expression and were classified as *cis*, whereas genes located more than 1 Mb apart or on different chromosomes were classified as *trans*. Among all selected interactions, genes identified as transcription factors or cofactors by the PigGTEx consortium [22] were annotated accordingly. The resulting networks were visualized and further analyzed using Cytoscape v3.10.2 [36].

The resulting networks were subjected to pathway enrichment analysis using the GO knowledgebase [37] and the R package ClusterProfiler v4.8.3 [38], using human annotation and the homologues of the identified genes according to the Ensembl database. The analysis was performed both on all genes in the network simultaneously and separately for each individual subnetwork.

To predict direct RNA-RNA interactions, the cDNA sequences of all pairs containing a lncRNA were extracted and submitted to IntaRNA v2.0 [39]. A seed length of 10 bp was used with a maximum gap of 0 within the seed. This analysis provided the aligning domains for each pair, as well as the associated free energy values. Interactions with a free energy lower than −30 kcal/mol were considered biologically plausible and selected as putative RNA-RNA interactions. Since IntaRNA may overestimate interactions by overlooking the accessibility of a given domain, RNAfold v2.7.2 was subsequently used to verify that the predicted binding sites are exposed in the lncRNA secondary structure.

## 3. Results

### 3.1. Classification of lncRNAs

Our study was based on an expression dataset comprising 16,063 genes profiled in blood samples from 255 commercial Duroc animals. Of these, 2563 genes were annotated as lncRNAs. A total of 7609 pairs of potentially interacting transcripts were detected by FEElnc, but only those classified by the software as best matches (2995 pairs) were selected for further analysis and are shown in Table 1. The complete list of predicted interactions is provided in Supplementary Table S2. Most lncRNAs did not share coding regions with other genes (76.8%) and were located antisense to their respective genes (58.9%). LncRNAs were evenly distributed between upstream and downstream regions of other genes, with downstream lncRNAs more frequently located on the same strand, whereas upstream lncRNAs were more commonly antisense. LncRNAs overlapping genic regions predominantly overlapped exons (9.3% of all pairs and 40.2% of genic pairs), marking them as potential candidates to produce silencing RNA molecules, as explained by Wucher et al. [34] in their breakdown of the FEElnc classification [34]. For more information about the categorization and meaning of each label, refer to Wucher et al. [34].

### 3.2. Gene Co-Expression Networks and Their Association with Immune Traits

**Figure 1:**
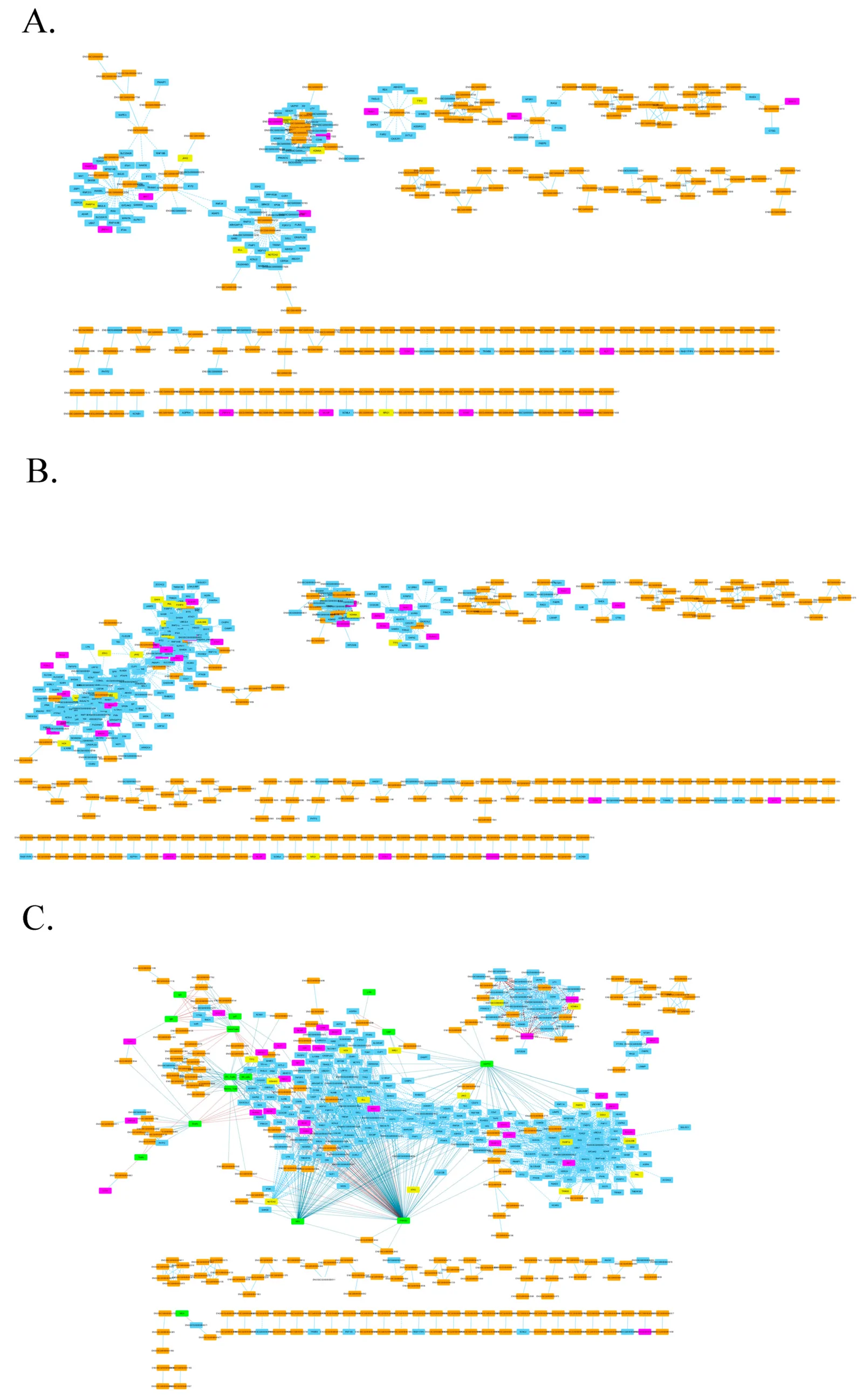
Networks depicting direct correlations between lncRNA, genes, and immune traits. Orange nodes represent lncRNA, blue nodes protein-coding genes, pink nodes transcription factors, yellow nodes transcription cofactors and green nodes immune traits. Edge colours indicate the sign of the correlation (blue, positive; red, negative), and edge line types indicate relative gene location, with dashed lines representing *trans* relative annotation and solid lines representing *cis*-relative annotation. Network (**A**) depicts direct correlations between lncRNAs and genes with a correlation weight greater than 0.9. Network (**B**) includes interactions shown in Network (**A**), with the addition of direct correlations (weight > 0.9) among genes present in Network (**A**). Network (**C**) depicts all interactions in Network (**B**), along with direct correlations with immune traits (weight > 0.3).

Networks depicting the PCIT-derived interactions among the expression levels of lncRNA, co-expressed genes, and immune traits are shown in Figure 1. Networks were constructed including lncRNA plus those genes directly correlated with them with |r| > 0.9, as well as the correlations (weight > 0.9) among themselves, and finally the immune traits correlating with selected genes with |r| > 0.3. A lower threshold for expression–phenotype associations was chosen due to the polygenic nature of immune traits, in which multiple gene expression levels are expected to contribute to the final phenotype. Therefore, correlations were expected to be lower than those observed between gene expression levels.

**Figure 2:**
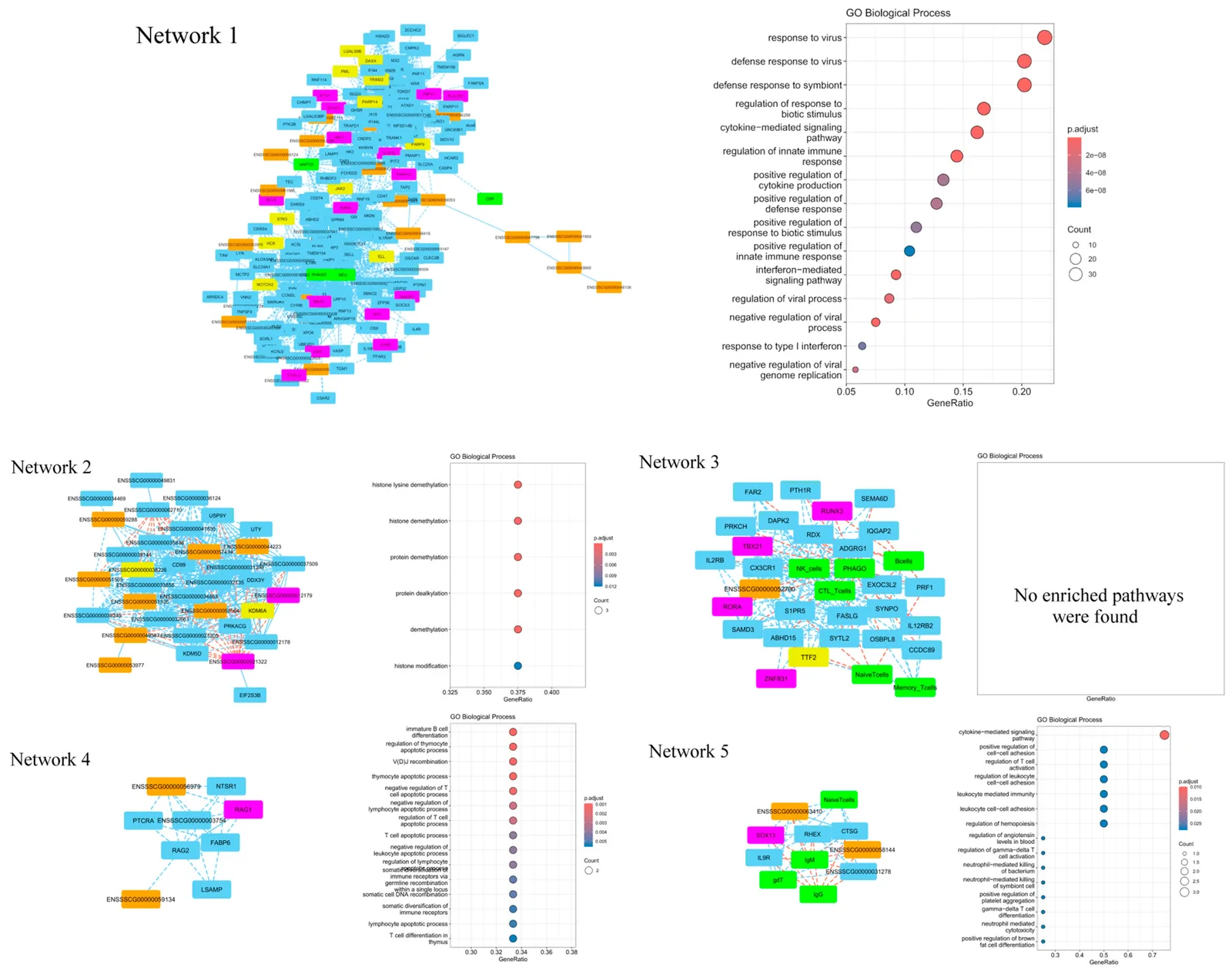
Independent networks containing more than four functionally characterized genes, with some of them identified in the GO database. Networks are ordered by the number of constituent genes. Each network is shown on the left, with the corresponding enriched pathway analysis displayed on the right. Orange nodes represent lncRNAs; blue nodes, protein-coding genes; pink nodes, transcription factors; yellow nodes, transcription cofactors; and green nodes, immune traits. Edge colour indicates correlation sign (blue, positive; red, negative) and edge line type denotes relative gene location (dashed line, *trans*; solid line, *cis*).

A total of 2637 direct correlations were selected as relevant, containing the expression levels of 187 lncRNAs. In addition, 473 correlations were identified between gene expression levels and immune traits. Most direct gene–gene correlations were positive, with only 44 negative interactions. Similarly, most gene–gene correlations were found in *trans*, with only 289 being in *cis*. Several genes identified in the correlation network showed potential transcription factor activity, with 29 described as transcription factors and 15 as transcription cofactors out of the 484 conforming genes. A total of 16 independent networks containing four or more genes were identified (Figure 1B), of which five contained more than four functionally characterized genes and were eligible for pathway analyses. These independent networks are shown in Figure 2 and are ranked in decreasing order according to the number of genes they contain. The full list of genes in each independent network can be found in Supplementary Table S3.

Limited overlap was found between the PCIT-derived correlations and the relative annotation. Only 32 directly correlated gene pairs were classified as potentially related by Feelnc. All pairs were intergenic, predominantly located on the same strand (29 pairs) and in a downstream orientation (23 pairs).

A total of 433 direct correlations between genes and immune traits were identified, with more than half (281) involving lncRNAs. Unlike correlations between genes, most correlations involving immune traits were positive (83%). The immune trait with the highest number of correlated genes was phagocytic intake (156 correlations), followed by neutrophil count (63 correlations). Both leukocyte count and mean corpuscular hemoglobin were each correlated with only a single gene. Table 2 presents a summary of the correlations found in the present networks for each analyzed trait.

Enrichment analyses were first performed on all 484 genes identified in the networks jointly. The analysis detected a total of 442 enriched biological terms in the Gene Ontology database, of which 278 were related to immunity. The full list of GO terms from both the joint analysis and each independent network can be found in Supplementary Table S4. The most representative biological process was defence response to virus, with 35 genes (30 of them from the largest independent network), followed by defence response to symbiont (also 35 genes). Other remarkable biological processes were interferon-mediated signalling pathway (17 genes), regulation of innate immune response (27 genes) and leukocyte mediated immunity (26 genes).

Most of the biological processes identified using the full set of genes overlapped with those found in the largest independent network, Network 1 (Figure 2). This network was strongly associated with phagocytic capacity with 116 of the 225 genes showing direct correlations, neutrophil count (57 correlations) and haptoglobin levels (37 correlations). Considering this, we next focused on the GO terms enriched by the smaller independent networks.

Network 2 (Figure 2) contained the genes *KDM5D*, *KDM6A* and *UTY*, and seven different lncRNAs. Functional enrichment analyses indicated that these genes were involved in histone demethylation, with no direct association with immune traits.

Network 3, containing a single lncRNA (ENSSSCG00000052700) and 26 annotated genes, including the transcription factor *RORA*, showed no significantly enriched terms. Interestingly, all 27 genes in this network were correlated with multiple immune traits. The strongest association was with CTL cells, which were correlated with all 27 genes, followed by natural killer cells and phagocytic capacity, each associated with 25 genes. Naïve T cells, memory T cells and B cells also presented correlations with several genes of this network.

Network 4 included genes such as *RAG1*, *RAG2* and *PTCRA*, and was associated with biological processes related to B cell differentiation, V(D)J recombination, and T cell apoptotic processes (Figure 2). This network contained the lncRNAs ENSSSCG00000059134 and ENSSSCG00000056979. However, none of the genes in this network correlated with any immune trait.

The final independent network, Network 5, included genes *ENSSSCG00000031278*, *CTSG*, *IL9R*, *SOX13* and *RHEX* and the lncRNAs ENSSSCG00000058144 and ENSSSCG00000063410. It was associated with several biological processes involving cytokine-mediated signalling pathway, regulation of T cell activation, leukocyte activation and cell adhesion and hemopoiesis (Figure 2). All seven genes in this network correlated with four immune traits: IgG, IgM, naïve T cells and γδ T cells.

### 3.3. RNA-RNA Interaction Analysis

**Figure 3:**
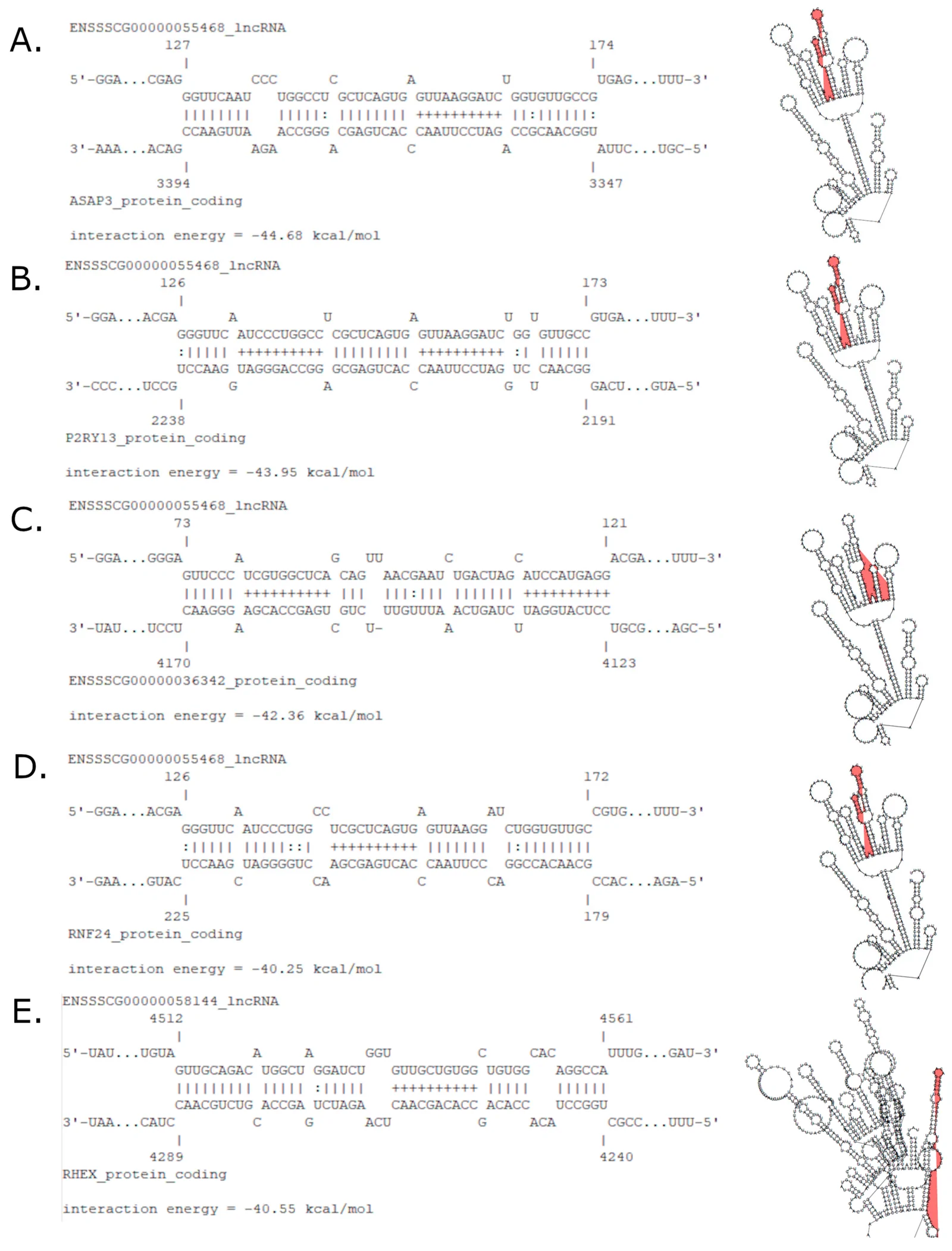
Putative RNA-RNA interactions showing the alignment of the interacting domain (**left**) and the lncRNA secondary structures with their interacting regions in red (**right**). The depicted pairs are (**A**) lncRNA ENSSSCG00000055468 paired with the gene *ASAP3* with interaction energy of −44.68 Kcal/mol; (**B**) lncRNA ENSSSCG00000055468 paired with the gene *P2PY13* with interaction energy of −43.95 Kcal/mol; (**C**) lncRNA ENSSSCG00000055468 paired with the gene ENSSSCG00000036342 with interaction energy of −42.36 Kcal/mol; (**D**) lncRNA ENSSSCG00000053468 paired with the gene *RNF24* with interaction energy of −40.25 Kcal/mol; and (**E**) lncRNA ENSSSCG00000058144 paired with the gene *RHEX* with interaction energy of −40.55 Kcal/mol.

All correlated pairs between lncRNAs and other genes were considered potential candidates for RNA-RNA interactions. According to IntaRNA, 178 pairs were predicted to have putative interactions. Of particular interest were 13 interactions involving genes from the previously identified independent networks that also shared direct correlations with the same immune traits. After assessing domain accessibility with RNAfold, five interactions, involving two different lncRNAs, remained (Figure 3).

From the main network, four of these putative interactions were between the lncRNA ENSSSCG00000055468 and the genes *ASAP3*, ENSSSCG00000036342, *RNF24* and *P2RY13*, all of which were directly correlated with phagocytic activity. In this lncRNA, all interacting domains were near each other, between positions 73 and 174 bp, with similar free energies around −42 kcal/mol and ~25 perfectly aligning nucleotides, suggesting a strong irreversible union.

The last putative interaction was found between the lncRNA ENSSSCG00000058144 and the gene *RHEX* of Network 5, with both genes directly correlated to γδ T cells, naïve T cells, IgG and IgM. The interacting domain was located between positions 4512 and 4561 bp and attached to the 5’UTR of *RHEX*, indicating a putative regulatory role. The interaction contained 30 perfectly aligning nucleotides with an interaction energy of −40.55 kcal/mol, also indicating a potential irreversible interaction.

## 4. Discussion

In this study, we analyzed whole-blood RNA-seq data from 255 commercial Duroc pigs to identify previously annotated lncRNAs associated with immunocompetence. The lack of a universal classification system for lncRNAs [40,41,42] complicates their annotation and interpretation. To address this, we used FEELnc for relative annotation and classification, which allows the categorization of lncRNAs based on genomic context and the prediction of antisense RNA-RNA interactions and divergent promoters [34]. Consistently with previous studies, most identified lncRNAs were intergenic [14,43] and were expressed at lower levels than protein-coding genes [43,44,45].

From the expressed lncRNAs, we constructed coexpression networks and assessed correlations with immune traits. As reported in previous studies [46,47], such networks are often complex and require careful interpretation. To improve interpretability, we restricted our analysis to genes with a high degree of coexpression with lncRNAs and genes strongly co-expressed with the previous set. The vast majority of correlations were positive. This may reflect the diverse regulatory functions of lncRNAs, which can act as positive regulators of gene expression [48], as well as coordinated regulation of lncRNAs and protein-coding genes by common transcription factors or signalling pathways [49,50].

We initially expected to identify several lncRNAs with suppressive effects on mRNA expression, particularly among those annotated by FEELnc as antisense and overlapping transcripts. However, the antisense and overlapping genomic classification of a lncRNA does not necessarily imply a suppressive regulatory effect. Antisense lncRNAs can exert either positive or negative regulatory effects on gene expression, depending on the genomic context and the molecular mechanisms involved [51]. Thus, the predominance of positive correlations observed in our analysis is not necessarily unexpected, even for lncRNAs with antisense and overlapping genomic annotations.

Only limited overlap was observed between the coexpression networks and FEELnc relative annotation. Although several *cis* correlations were detected, the genomic window used by FEELnc (1 kb) is much smaller than the 1 Mb window used in our *cis*/*trans* analysis, which may partly explain the limited overlap between the two approaches. In addition, the stringent correlation threshold used in the coexpression analysis (|0.9|) may have further reduced the number of shared lncRNA–gene pairs. This threshold was chosen to improve rigour and limit false positives, following the approach used by Deng et al. [43] in similar analyses. Only 32 lncRNA–gene pairs were retained in both analyses, all of which corresponded to intergenic lncRNAs. The limited representation of antisense and overlapping lncRNAs among these shared pairs may reduce the likelihood of detecting direct silencing interactions and may therefore partly contribute to the predominance of positive correlations observed in our coexpression analysis.

We discovered several independent networks, with one remarkable main network comprising 18 different lncRNAs and 206 additional genes, among which were several transcription factors and cofactors. Notably, many of these genes were associated with immune-related biological processes, including *ZNFX1* and *IRF7*, which are involved in response to bacteria [52,53]. Remarkably, *MyD88*, which is known to interact with *IRF7*, was also found as part of the network [54]. Additional protein-coding genes encoding transcription factors involved in B-cell function and interleukin-4 production included *BCL6*, *ETV7*, and *SPI1* [55,56,57], while *STAT1* and *STAT2*, involved in interferon production [58,59], were also present. The presence of these transcription factors was reflected in the enrichment analysis, which revealed several immunity-related GO terms consistent with the immune traits correlated with this network, particularly phagocytic capacity.

Notably, several biological processes associated with virus response were among the most enriched in the main candidate lncRNA-mediated coexpression network in blood, consistently with results reported by Shi et al. [17] in spleen. In their study, Shi et al. [17] characterized lncRNAs in the spleen of Meishan pigs and observed enrichment of immune processes related to virus response and type I interferon signalling. These immune pathways are known to be regulated by lncRNAs in humans, with involvement in pattern recognition receptor activity [60] and interferon production via IFN-activated JAK-STAT signalling [61]. One of the central genes identified in the Meishan spleen network, *DHX58*, was also present as a hub gene in our pig blood network, where it showed direct correlations with 46 genes, including one lncRNA (ENSSSCG00000052056). *DHX58* is a member of the RIG-I-like receptor-associated family of pattern-recognition receptors involved in innate immune responses, particularly antiviral defence through the induction of inflammatory mediators in M1 macrophages and the modulation of RIG-I/MDA5-mediated type I interferon responses [62,63]. Moreover, *DHX58* has been proposed as a candidate gene for breeding more robust animals, as an SNP within this gene was associated with immune traits such as blood cell distribution in pigs [64]. Although *IFIT1*, another core gene in the spleen coexpression network of Shi et al. [17], was not present in our blood coexpression network, other members of the IFIT family were, specifically *IFIT2*, *IFIT3* and *IFIT5*. Moreover, genes encoding the transcription factors *STAT1* and *STAT2*, also involved in interferon-mediated antimicrobial responses [65], were identified in the network. The presence of *STAT* family members is noteworthy given the well-characterized lncRNA-mediated regulation of *STAT3* in human cancer [66], where *STAT3* expression and activity are modulated by several lncRNAs involved in apoptosis, metastasis and drug resistance [67]. However, to our knowledge, direct and well-characterized lncRNA-mediated regulation of *STAT1* and *STAT2* in humans remains limited, although post-transcriptional regulation of these genes by miRNAs has been reported [68,69].

In this context, the detection of *STAT1* and *STAT2* in our network, together with the relationships between *IFIT1* and *STAT1* reported by Shi et al. [17] and the direct correlations observed with several lncRNAs, may suggest lncRNA-associated regulatory patterns involving components of the STAT signalling pathway in pigs. Taken together, the results of both studies suggest that spleen and blood tissues may share interferon-related immunoregulatory architectures potentially influenced by lncRNAs associated with key antiviral mediators such as *DHX58* and STAT family members, although the specific regulatory lncRNAs appear to differ between tissues.

The most relevant lncRNA in our main network was ENSSSCG00000055468. Notably, this was the only lncRNA present in a coexpression network identified in a previous study [24] as putatively regulated by a transcriptional regulatory hotspot on chromosome 6, rs1107483072. This variant was associated with the expression of 44 out of the 224 genes in the main coexpression network, being the most significant variant of the eQTL in 22 of the 44 cases. The lncRNA ENSSSCG00000055468 was predicted to share a putative interaction domain with four different genes, all located in the same region of its secondary structure. All interacting genes, *ASAP3*, ENSSSCG00000036342, *RNF24* and *P2RY13*, were directly correlated with phagocytic capacity. Although no studies have directly linked *ASAP3*, *RNF24* and *P2RY13* to phagocytic activity, these genes encode proteins involved in membrane-associated processes [70,71,72], which are often important for cellular functions underlying phagocytosis. Moreover, all four genes share a putative interacting domain with ENSSSCG00000055468, all located within UTR regions: 3’ UTR for *ASAP3*, ENSSSCG00000036342 and *P2RY13* and 5’ UTR for *RNF24*. Together with the observed positive expression correlations, these findings are consistent with a potential regulatory role of this lncRNA. Further experimental validation is required to confirm these interactions and their association with phagocytosis function.

The rest of the candidate lncRNA-mediated networks identified in the blood transcriptome also offered some interesting findings regarding the role of lncRNA in pig immunity. The third network contained several genes highly correlated with T cell subpopulation distribution with a single lncRNA (ENSSSCG00000052700). Notably, the *RORA* gene, which encodes the transcription factor RORA, was present in this network. In a previous study conducted in the same population [24], in silico analysis of candidate regulatory variants associated with QTLs for helper and memory T-cell proportions identified several polymorphisms that potentially affect RORA binding sites, suggesting an important role for this transcription factor in regulating the proportions of different T-cell populations. Additionally, *IL12RB2*, *PRKCH*, *RUNX3* and *TBX21*, all genes related to regulation of CD4+ T cells [73,74,75,76], were also found in the third network.

The fifth candidate lncRNA-mediated network presented the only additional putative RNA-RNA interaction between the lncRNA ENSSSCG00000058144 and the gene *RHEX*, which is involved in erythrocyte differentiation [77]. Similar to the other domains detected in the main network, the interacting region was located in the 5’ UTR region and was of similar length. The most remarkable feature of this network was the abundance of direct correlations to immune phenotypes. All nodes were associated with IgG and IgM, naïve and γδ T cells, and contained several immune-related candidate genes such as *CTSG*, *IL9R* and *SOX13* [78,79,80]. Of particular interest is the presence of *SOX13*, whose role as a γδ T-cell differentiation factor had already been highlighted [27]. Although pathway analysis results were heterogeneous, all enriched terms were related to immune processes. Further research on this gene cluster could provide insights into immune mechanisms, specifically by experimentally validating the predicted interaction between ENSSSCG00000058144 and *RHEX*.

Finally, it is important to acknowledge three main limitations of the present study. First, although we used the lncRNA annotation tool FEElnc, our objective was not to perform a de novo lncRNA annotation. This study was motivated by the large number of hotspot-regulated lncRNAs found in a previous study [24]. Accordingly, the main objective was to investigate lncRNA-regulated networks using the same annotated dataset. Several studies have performed lncRNA annotation in pigs [22,43], with some of them being incorporated in the current genome annotation [81]. Therefore, we considered the available annotation of lncRNA in the porcine genome to be sufficient for the purposes of this study. The second major limitation is that all detected interactions should be considered preliminary and require experimental validation, as bioinformatic tools are not sufficient to fully assess a putative interaction. Third, the transcriptomic profile is known to be highly variable across population, tissue or developmental stage. In our study a commercial 60-day-old Duroc population was used, as such, we cannot discard breed- and age-specific network architectures that should be validated in other populations and developmental stages.

In conclusion, this study used RNA-seq data to explore the potential role of lncRNAs in immune regulation in peripheral blood. We constructed coexpression networks that revealed direct correlations between gene expression profiles and immune phenotypes, identifying several immune-related networks enriched in candidate genes for viral response and B and T cell differentiation. From these candidates, we predicted putative RNA-RNA interactions. Further research is needed to validate these interactions and elucidate the regulatory mechanisms mediated by lncRNAs. Overall, all these data contribute to expanding our understanding of porcine gene expression regulation and provide novel insights into the mechanisms that underlie immune-related phenotypes.

## Figures and Tables

**Table 1:** Characterization of most relevant lncRNAs in the porcine blood transcriptome based on their closest annotated transcript.

Direction	Genomic Location	Type	Relative Location	Occurences
Antisense	Genic	Containing	Exonic	107
			Intronic	93
		Nested	Exonic	62
			Intronic	25
		Overlapping	Exonic	279
			Intronic	10
	Intergenic	Convergent	Downstream	439
		Divergent	Upstream	749
Sense	Genic	Containing	Exonic	8
			Intronic	49
		Nested	Exonic	4
			Intronic	47
		Overlapping	Exonic	9
			Intronic	1
	Intergenic	Convergent	Downstream	711
		Divergent	Upstream	402

**Table 2:** Number of direct correlations of immune traits with genes included in the candidate lncRNA-mediated networks, by gene type.

Trait	Correlated Genes	lncRNA	Protein-Coding Genes	Transcription Factors	Transcription Cofactors
Immunoglobulin G	9	4	4	1	0
Immunoglobulin M	10	4	4	2	0
C-reactive protein	5	2	2	0	1
Haptoglobin	41	6	31	3	1
Mean corpuscular hemoglobin	1	0	1	0	0
Neutrophil count	63	6	50	4	3
Lymphocyte count	1	1	0	0	0
% Phagocytic cells	156	19	120	13	4
T cells	3	3	0	0	0
B cells	18	7	8	3	0
Natural Killer cell	33	7	21	4	1
Cytotoxic T cells	43	16	22	4	1
γδ T cells	11	5	4	2	0
Memory T cells	20	13	6	0	1
Naïve T cells	19	9	8	1	1

## Data Availability

The raw sequence data presented in this study are openly available in the SRA repository with the Bioproject accession code PRJNA1267984. The data of Significant associations detected through eGWAS presented in this study are openly available in CORA.RDR repository at: https://doi.org/10.34810/data2296.

## Supplementary Materials

The following supporting information can be downloaded at https://www.mdpi.com/article/10.3390/cells15181681/s1. Table S1: Descriptive statistics of phenotypes used in the present study. PBMC stands for Peripheral Blood Mononuclear Cells; Table S2: Output of FEElnc relative annotation; Table S3: Genes located in each of the independent networks; Table S4: Pathway represented in networks.

## Abbreviations

The following abbreviations are used in this manuscript:

lncRNA	Long non-coding RNAs
PBMC	Peripheral mononuclear blood cell
IgG	Immunoglobulin G
IgM	Immunoglobulin M
CRP	C-reactive protein
PCIT	Partial correlation and information theory
